# Supplementary material for: Neisseria meningitidis filamentous phage MDA promotes colonisation by selecting hyperadhesive pili variants
Source: Nat Commun. 2025 Dec 20;17:744. doi: 10.1038/s41467-025-67441-w (PMC12819557; doi:10.1038/s41467-025-67441-w)
Supplement: Supplementary file 1 — Supplementary Information [file 41467_2025_67441_MOESM1_ESM.pdf]

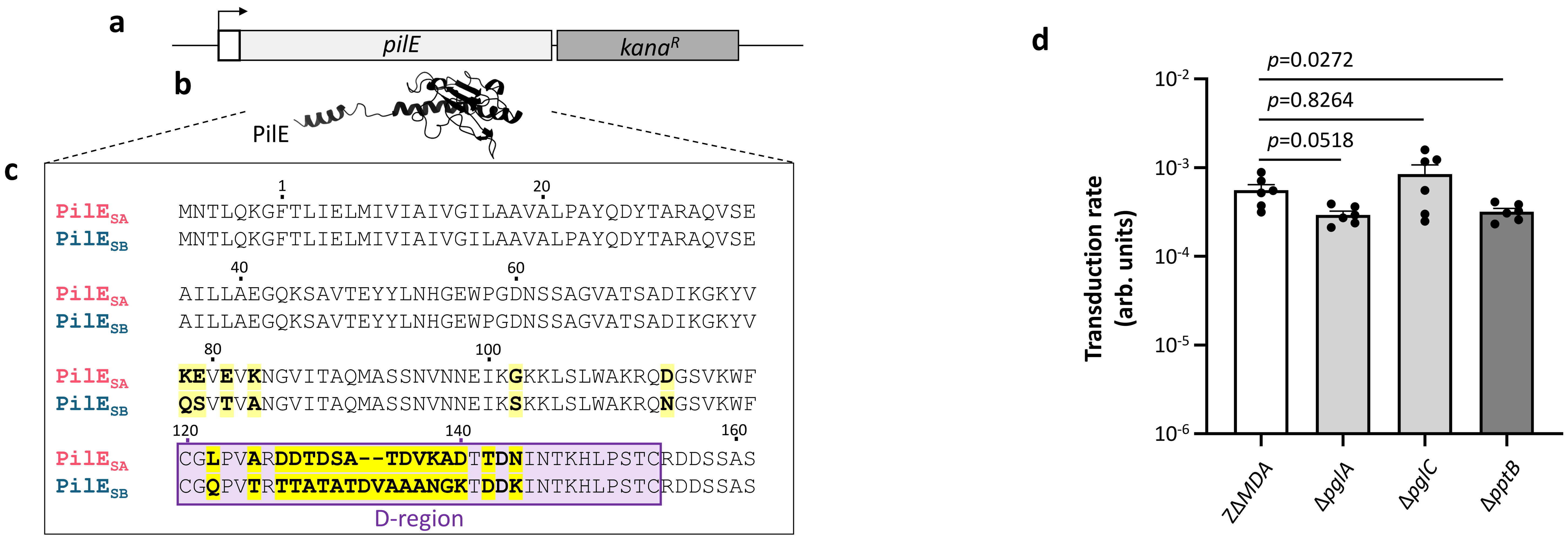

Supplementary Figure 1. **a** Schematic representation of the transcriptional fusion between the class I *pilE* gene and the kanamycin resistance gene. **b** PilE structure from subunit B of PDB: 5KUA structure. **c** Protein sequence alignment of the PilESA, and PilESB variants is shown with amino acid differences highlighted in yellow and the D-region in purple. **d** Transduction rate of the ZΔ*MDA* strains and their derivatives mutated in genes responsible for the O-linked protein glycosylation system (*pglA* and *pglC*) and the transfer of phosphoglycerol to pilin (*pptB* for pilin phosphotransferase B) (arb. units: arbitrary unit). Experiments were performed three times in duplicate (n=3). Statistical analyses were performed using a Brown-Forsythe and Welch ANOVA test (two-sided) with Dunnett's correction, and data were expressed as mean ± SEM. Source data are provided as a Source Data file.

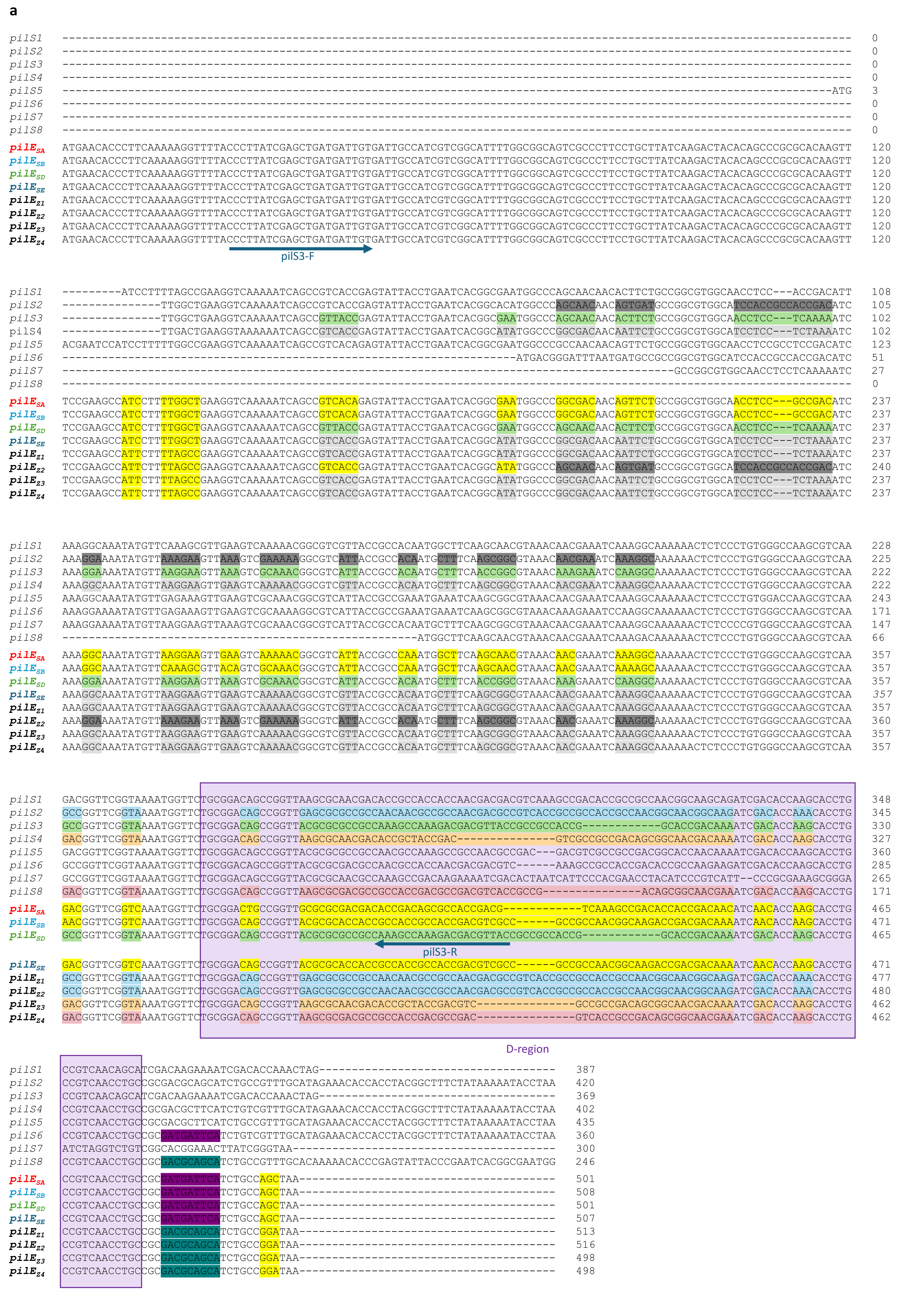

Supplementary Figure 2.

b

```
PilS1     -------------------------------------------ILLAEGQKSAVTEYYLNHGEWPSNNTSAGVATS-TDIKGKYVQSVEVKNGVVTATMASSNVNNEIKGKKLSLWAKRQ   76
PilS2     ---------------------------------------------LAEGQKSAVTEYYLNHGTWPSNNSDAGVASTATDIKGKYVKEVKVEKGVITATMLSSGVNNEIKGKKLSLWAKRQ   75
PilS3     ---------------------------------------------LAEGQKSAVTEYYLNHGEWPSNNTSAGVATS-SKIKGKYVKEVKVANGVITATMLSTGVNKEIQGKKLSLWAKRQ   74
PilS4     ---------------------------------------------LTEGKKSAVTEYYLNHGIWPGDNNSAGVASS-SKIKGKYVKEVEVKNGVVTATMLSSGVNNEIKGKKLSLWAKRQ   74
PilS5     ---------------------------------------MTNPSFLAEGQKSAVTEYYLNHGEWPANNSSAGVATSASDIKGKYVEKVEVANGVITAEMKSSGVNNEIKGKKLSLWTKRQ   81
PilS6     ---------------------------------------------------------------MTGFNDAAGVASTATDIKGKYVEKVEVAKGVITAEMKSSGVNKEIQGKKLSLWAKRQ   57
PilS7     ----------------------------------------------------------------------AGVATS-SKIKGKYVKEVKVANGVITATMLSSGVNNEIKGKKLSPWAKRQ   49
PilS8     --------------------------------------------------------------------------------------------------MASSNVNNEIKDKKLSLWAKRQ   22

PilE_SA   MNTLQKGFTLIELMIVIAIVGILAAVALPAYQDYTARAQVSEAILLAEGQKSAVTEYYLNHGEWPGDNSSAGVATS-ADIKGKYVKEVEVKNGVITAQMASSNVNNEIKGKKLSLWAKRQ   119
PilE_SB   MNTLQKGFTLIELMIVIAIVGILAAVALPAYQDYTARAQVSEAILLAEGQKSAVTEYYLNHGEWPGDNSSAGVATS-ADIKGKYVQSVTVANGVITAQMASSNVNNEIKSKKLSLWAKRQ   119
PilE_SD   MNTLQKGFTLIELMIVIAIVGILAAVALPAYQDYTARAQVSEAILLAEGQKSAVTEYYLNHGEWPSNNTSAGVATS-SKIKGKYVKEVKVANGVITATMLSTGVNKEIQGKKLSLWAKRQ   119
PilE_SE   MNTLQKGFTLIELMIVIAIVGILAAVALPAYQDYTARAQVSEAILLAEGQKSAVTEYYLNHGIWPGDNNSAGVASS-SKIKGKYVKEVEVKNGVVTATMLSSGVNNEIKGKKLSLWAKRQ   119
PilE_Z1   MNTLQKGFTLIELMIVIAIVGILAAVALPAYQDYTARAQVSEAILLAEGQKSAVTEYYLNHGIWPGDNNSAGVASS-SKIKGKYVKEVEVKNGVVTATMLSSGVNNEIKGKKLSLWAKRQ   119
PilE_Z2   MNTLQKGFTLIELMIVIAIVGILAAVALPAYQDYTARAQVSEAILLAEGQKSAVTEYYLNHGIWPSNNSDAGVASTATDIKGKYVKEVKVEKGVITATMLSSGVNNEIKGKKLSLWAKRQ   120
PilE_Z3   MNTLQKGFTLIELMIVIAIVGILAAVALPAYQDYTARAQVSEAILLAEGQKSAVTEYYLNHGIWPGDNNSAGVASS-SKIKGKYVKEVEVKNGVVTATMLSSGVNNEIKGKKLSLWAKRQ   119
PilE_Z4   MNTLQKGFTLIELMIVIAIVGILAAVALPAYQDYTARAQVSEAILLAEGQKSAVTEYYLNHGIWPGDNNSAGVASS-SKIKGKYVKEVEVKNGVVTATMLSSGVNNEIKGKKLSLWAKRQ   119

PilS1     DGSVKWFCGQPVKRNDTATTNDDVKADTAANGKQIDTKHLPSTA-STRKSTPN---------------   128
PilS2     AGSVKWFCGQPVERAANNAANDAVTAATANGNGKIDTKHLPSTC-RDAASAVCIETPPTAFYKNT---   139
PilS3     AGSVKWFCGQPVTRAAKAK----DDVTAATGTDKIDTKHLPSTA-STRKSTPN---------------   122
PilS4     DGSVKWFCGQPVKRNDTA-----TDVAADSGNDKIDTKHLPSTC-RDASSVVCIETPPTAFYKNT---   133
PilS5     DGSVKWFCGQPVTRAANAKAANADD-VAADGTNKIDTKHLPSTC-RDASSAVCIETPPTAFYKNT---   144
PilS6     AGSVKWFCGQPVTRDANATNDD--VKAATDTAKKIDTKHLPSTC-RDDSSVVCIETPPTAFYKNT---   119
PilS7     AGSVKWFCGQPVTRNAKADKK-------------IDTNHSHEPTSRHSRESGNLGLSARKLIG-----   99
PilS8     DGSVKWFCGQPVKRDAATDAD-----VTADSGNEIDTKHLPSTC-RDAASAVCTKTPE--YYPNHGEW   82

PilE_SA   DGSVKWFCGLPVARDDTDSA----TDVKADTTDNINTKHLPSTC-RDDSSAS----------------   166
PilE_SB   NGSVKWFCGQPVTRTTATATDV--AAANGKTDDKINTKHLPSTC-RDDSSAS----------------   169
PilE_SD   AGSVKWFCGQPVTRAAKAK----DDVTAATGTDKIDTKHLPSTC-RDDSSAS----------------   166
PilE_SE   DGSVKWFCGQPVTRTTATATDV--AAANGKTDDKINTKHLPSTC-RDDSSAS----------------   168
PilE_Z1   AGSVKWFCGQPVERAANNAANDAVTAATANGNGKIDTKHLPSTC-RDAASAG----------------   170
PilE_Z2   AGSVKWFCGQPVERAANNAANDAVTAATANGNGKIDTKHLPSTC-RDAASAG----------------   171
PilE_Z3   DGSVKWFCGQPVKRNDTA-----TDVAADSGNDKIDTKHLPSTC-RDAASAG----------------   165
PilE_Z4   DGSVKWFCGQPVKRDAAT-----DADVTADSGNEIDTKHLPSTC-RDAASAG----------------   165
                        D-region
```

Supplementary Figure 2. **a,b** Sequence alignment of DNA (a) and amino acid translation (b) of the eight silent *pilS* loci of the reference strain Z5463, and $pilE_{SA}$, $pilE_{SB}$, $pilE_{SD}$ and $pilE_{SE}$, $pilE_{Z1}$, $pilE_{Z2}$, $pilE_{Z3}$, $pilE_{Z4}$ variants. Codon and amino acid differences are highlighted according to their correspondence with the specific *pilS*: upstream sequences of *pilE*/PilE are highlighted in grey, green, and pink for regions corresponding to *pilS2*, *pilS3* and *pilS4* respectively. Downstream regions in and near D-region of *pilE*/PilE are highlighted in blue, green, orange, and red, corresponding to *pilS2*, *pilS3*, *pilS4*, and *pilS8*, respectively. The very downstream region, close to the end, are highlighted in purple and dark green, corresponding to *pilS6* and *pilS8*, respectively. The *pilE* codon variations and their corresponding amino acids that are not associated with a specific *pilS* are highlighted in yellow. Sequences were aligned using Clustal-omega (1.2.4). Blue arrows on the DNA alignment indicate the positions of the primers used for the specific amplification of the *pilS3* variant by qPCR. The D-region of the PilE gene and proteins are shown in purple.

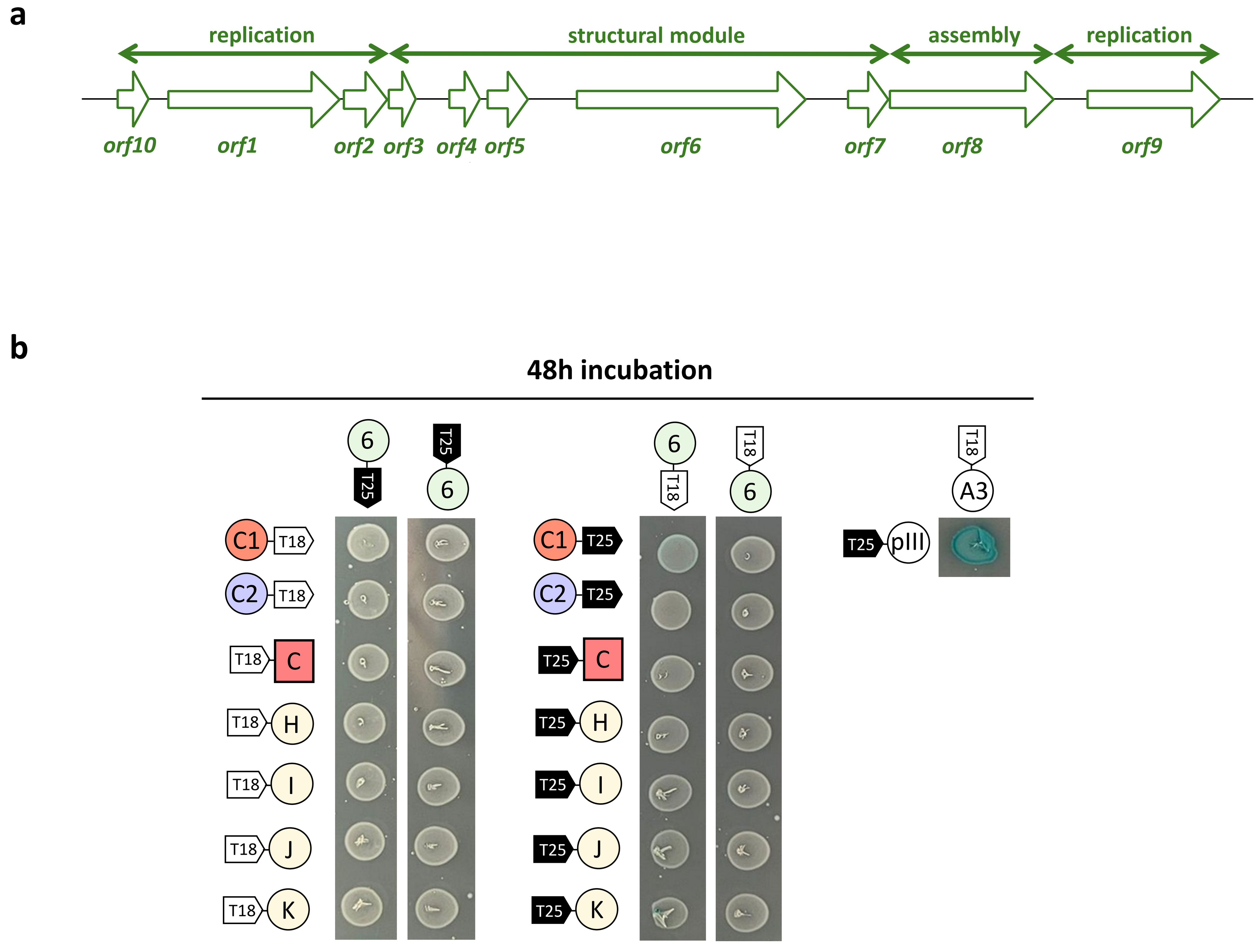

Supplementary Figure 3. **a** The MDAΦ genome has 10 Open Reading Frames: *orf10*, *orf1*, *orf2*, *orf9* are responsible for cytoplasmic phage replication, *orf3*, *orf4*, *orf5*, *orf6*, *orf7* are involved in the phage morphogenesis and *orf8* is involved in the phage assembly. **b** Interaction studies of the phage coat protein ORF6 with domains of pilus fibre proteins (circled: PilC1-Nter, PilC2-Nter, PilH, PilI, PilJ, PilK; framed: PilC-Cter). Bacterial two-hybrid assays were performed using the Oxi-Blue reporter strains producing the indicated protein domains fused to the T18 or T25 domain of the *Bordetella pertussis* adenylate cyclase. Bacteria were spotted on plates supplemented with IPTG and X-Gal and imaged after 48 hours of incubation. The interaction between the two fusion proteins is indicated by the blue colour. The interaction between pIII ($pIII_{M13}$) and TolA3 ($TolA3_{E. coli}$) is used as a positive control.

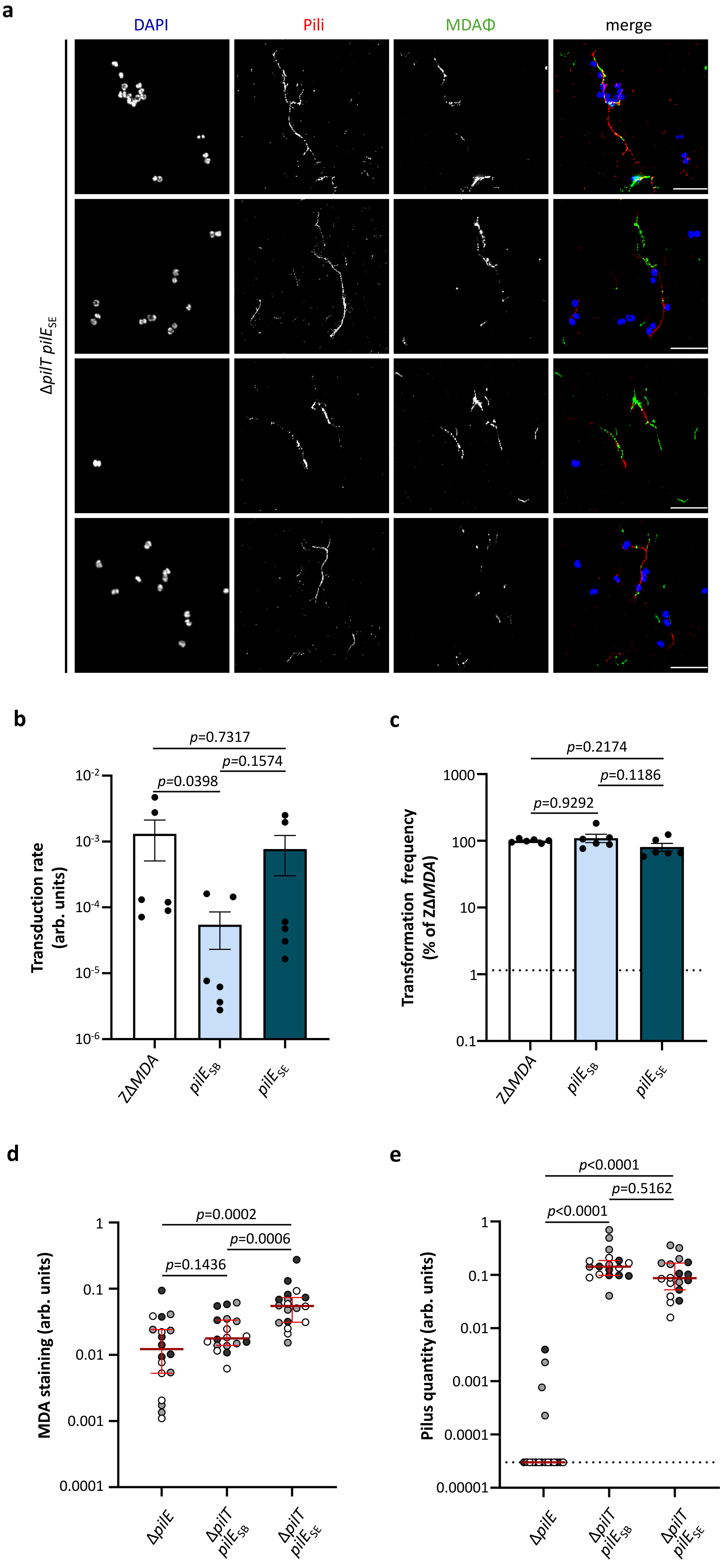

Supplementary Figure 4. **a** Complementary images from Fig. 4d. $MDA_{(orf6\text{-}aadA1)}$Φ binding to strains ZΔ*MDA* Δ*pilT pilE*$_{SE}$ was imaged using high-resolution STED microscopy. Bacteria were incubated with phages, washed, precipitated and immobilised on glass slides. Bacteria were revealed using DAPI (first column, DNA staining, blue), pili and phages were labelled with anti-PilE 20D9 antibody (second column, anti-pili, red) and anti-$MDA_{ORF4\_N\text{-}ter}$ antibody (third column, anti-phage, green). Bar = 50 µm. **b,c** Phenotypes of transduction (b) and transformation (c) for the strain ZΔ*MDA* and its derivatives expressing the $PilE_{SB}$ or $PilE_{SE}$ variants. Transduction results were expressed as transduction rates (arb. units: arbitrary unit) and transformation results were expressed as a percentage of that of ZΔMDA. Experiments were performed three times in duplicate (n=3). Statistical analyses were performed using an ordinary ANOVA test with Tukey correction, and data were expressed as mean ± SEM. The dotted line corresponds to the mean threshold for the appearance of spontaneous nalidixic acid-resistant clones. Source data are provided as a Source Data file. **d,e** MDA staining on strains expressing different PilE variants (d) and pili quantity of these strains (e) were estimated by immunofluorescence as the ratio of phage surface or pili surface labelling to bacterial surface labelling after phage precipitation. Statistical analysis was performed using a Brown-Forsythe and Welch ANOVA tests (two-sided) with Tamhane's T2 correction for d, Kruskal-Wallis test with Dunn's correction for e, and data were expressed as median with 95% CI. Each dot represents one image and images were taken from three different experiments (n=3), with dots coloured black, grey and white, respectively. The dotted line represents the detection threshold. Source data are provided as a Source Data file.

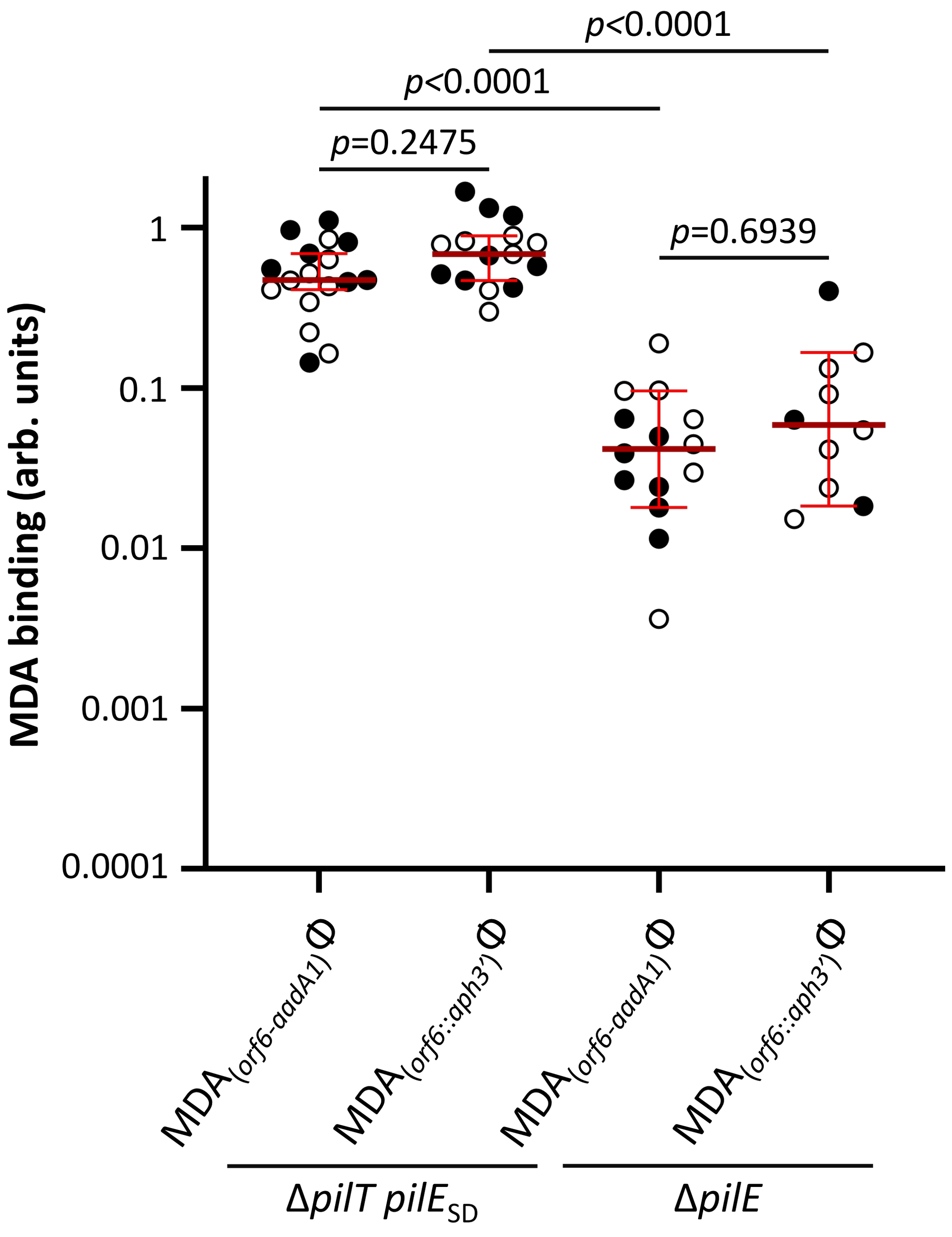

Supplementary Figure 5. **MDA$_{(orf6\text{-}aadA1)}$Φ and MDA$_{(orf6::aph3')}$Φ binding to strain ZΔ*MDA*Δ*pilT pilE*$_{SD}$ and the pili-deficient strain ZΔ*MDA* Δ*pilE*.** Binding was estimated by immunofluorescence as the ratio of phage surface labelling to bacterial surface labelling after phage precipitation (arb. Units: arbitrary unit). Statistical analysis was performed using a Brown-Forsythe and Welch ANOVA test (two-sided) with Tamhane T2 correction. Data were expressed as median with 95% CI. Each dot represents one image and images were taken from two different experiments (n=2), with dots colored black and white, respectively. Source data are provided as a Source Data file.

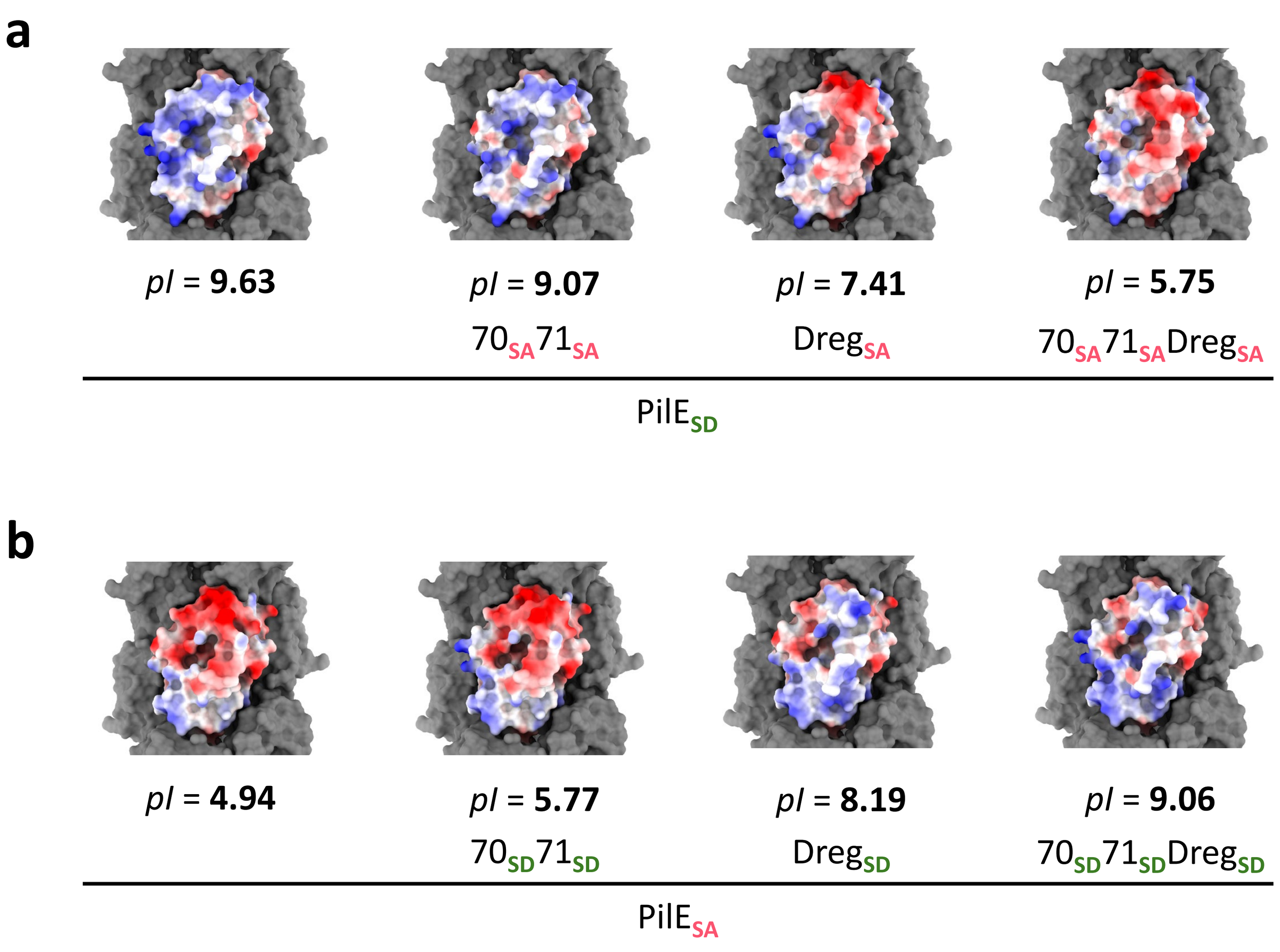

Supplementary Figure 6. **a,b** Reconstruction of pilus fibres from bacteria expressing the $PilE_{SA}$ and $PilE_{SD}$ variants and their derivative mutants in which amino acid 70-71 and/or the D-region (Dreg) have been swapped. The structures are based on the biological assembly 5KUA and PilE subunits obtained using alphafold-3. The structures were coloured using the coulombic electrostatic potential command of ChimeraX with default colouring ranging from red for negative potential through white to blue for positive potential. pI (isoelectric point) was determined using iep tool of Galaxy version 5.0.0.1 with initial parameters.

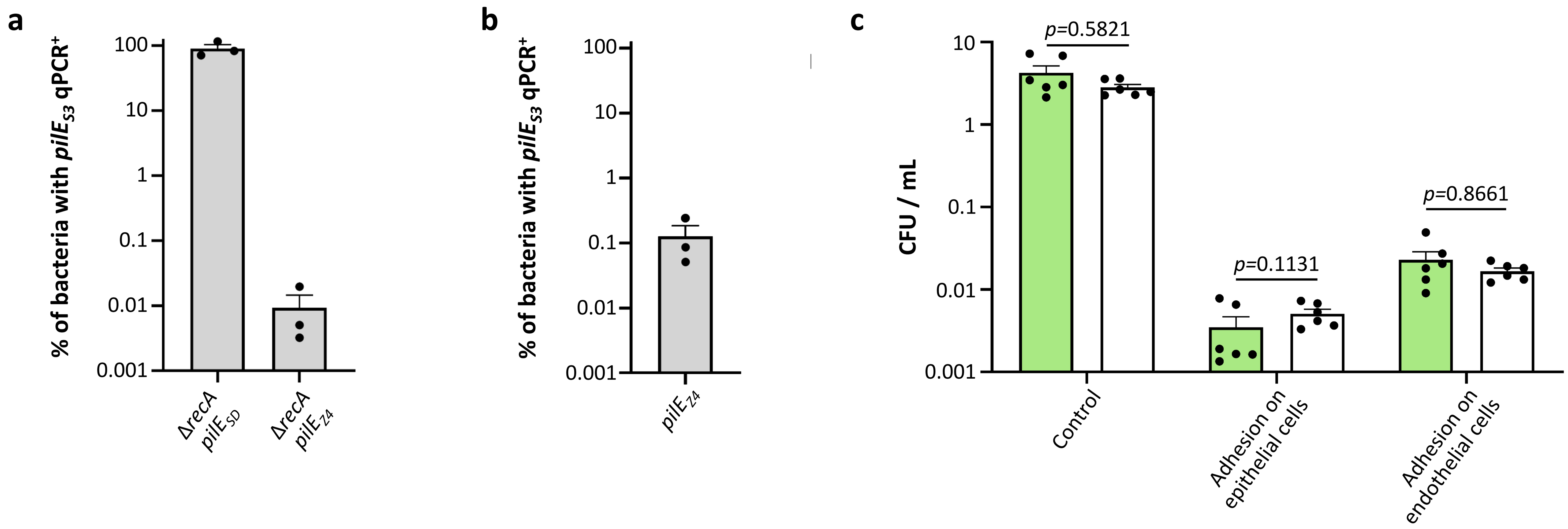

Supplementary Figure 7. **a, b** (related to Fig. 6 a-g**)** The proportion of bacteria expressing a $\mathrm{PilE_{S3}}$ variant was quantified by qPCR. In (a), clonal ZΔ*MDA* Δ*recA* $pilE_{SD}$ and ZΔ*MDA* Δ*recA* $pilE_{Z4}$ are the positive and negative control of this assay, respectively. In b, we determined the proportion of bacteria expressing a $\mathrm{PilE_{S3}}$ variant in the initial ZΔ*MDA* $pilE_{Z4}$ population. **c (**related to Fig. 7 a,b**)** Quantification of the number of bacteria of heterogeneous phage-infected bacterial population (green) and uninfected control population (white) after adhesion selection on epithelial cells or endothelial cells, or after growth in cell culture media. Experiments were performed three times in duplicate (n=3). Statistical analysis was performed using a 2-way ANOVA test with Sidak correction, and data were expressed as mean ± SEM. Source data are provided as a Source Data file.
