# Supplementary material for: Neisseria meningitidis filamentous phage MDA promotes colonisation by selecting hyperadhesive pili variants
Source: Nat Commun. 2025 Dec 20;17:744. doi: 10.1038/s41467-025-67441-w (PMC12819557; doi:10.1038/s41467-025-67441-w)
Supplement: Supplementary file 2 — Description of Additional Supplementary File [file 41467_2025_67441_MOESM2_ESM.pdf]

Description of Additional Supplementary Files

Supplementary data 1: Strains used in the study

Supplementary data 2: Primers used in the study

Supplementary data 3: Plasmids used in the study
